# Terminology of Lower Urinary Tract Symptoms. Helpful or Confusing ?

**DOI:** 10.1100/tsw.2009.9

**Published:** 2009-01-18

**Authors:** Sonia Vishwajit, Karl-Erik Andersson

**Affiliations:** Wake Forest Institute for Regenerative Medicine, Wake Forest University School of Medicine, Winston-Salem, USA

**Keywords:** overactive bladder, urgency, detrusor overactivity

## Abstract

An established standardized terminology is necessary for communication of scientific information, and for prevention of mistreatment and misdiagnosis. Terminology concerning the lower urinary tract has been much discussed; in particular, the meaning of terms like lower urinary tract symptoms (LUTS), urgency, frequency and nocturia, overactive bladder (OAB), and detrusor overactivity (DO). It is natural and desirable that all suggested definitions are subject to criticism, and it is important that discussions for improvement of the existing terminology continue.

## INTRODUCTION

The report of the Standardization Sub-committee of the International Continence Society (ICS) from 2002[1] has been one of the most cited documents in the field of lower urinary tract function and dysfunction. This may partly reflect the fact that uniform definitions are needed both for research purposes and for diagnosis and treatment of lower urinary tract LUT disorders, but also the fact that not everybody agrees with the definitions given[2]. It is obvious that linguistic ambiguity leads to a great deal of confusion and can be highly problematic when it pertains to medical terminology. For physicians/researchers, an established standardized terminology is necessary for communication of scientific information, and for prevention of mistreatment and misdiagnosis. It is also mandatory that the patients understand what the doctor is talking about[3]. In particular, the meaning of terms like lower urinary tract symptoms (LUTS), urgency, frequency and nocturia, overactive bladder (OAB), and detrusor overactivity (DO) have been extensively discussed[2,4,5,6,7]. Below, some aspects on the definitions and implications of the use of these terms will be briefly reviewed.

## LOWER URINARY TRACT SYMPTOMS (LUTS)

According to the ICS report[1], LUTS is defined as “the subjective indicator of a disease or change in condition perceived by the patient, caregiver or partner and may lead him/her to seek help from health care professionals. Symptoms may either be volunteered or described during the patient interview, are usually qualitative, and cannot be used to make a definitive diagnosis. LUTS can be divided into 3 groups (a) storage symptoms (b) voiding symptoms and (c) post micturition symptoms."

Digesu et al. performed a questionnaire study with 138 women attending urodynamic clinics in three different countries, testing the knowledge of six different LUTS: urgency urinary incontinence, stress urinary incontinence, daytime frequency, urgency, nocturia, and hesitancy[3]. Five possible explanations for the meaning of each symptom were given. The women (three groups based on nationality) then underwent urodynamic investigation using a standardized protocol. The correct given definitions and urodynamic diagnoses were compared between the three groups of women to check if the symptoms from which they suffered altered their correct understanding of the symptom definition. Of the 138 subjects, only 33 and 44% of the women knew the correct definitions of increased daytime frequency and nocturia, respectively. More than 20% of the women were unsure of the meaning of the terms that the clinicians might use, and less than 50% of women understood the LUTS terminology as defined by the ICS.

Since the answers to questionnaires are dependent on the patients' understanding of the questions asked, an explanation of the meaning of the terms used should be a prerequisite in order to obtain accurate information both in a clinical setting and in clinical trials. It is mandatory for correct diagnosis and management that both patient and physician have the same interpretation of the terms used to described the patient's disorder. A "passive" recording of the answers to questions the patients do not understand seems not only meaningless, but may lead to misdiagnosis and incorrect treatment.

In men, the term LUTS was introduced to separate male urinary symptoms from any implied symptom origin, such as the prostate. Even if there is still a strong association between male LUTS and the prostate, the paradigm has shifted to focus on the bladder[8]. LUTS in men typically occur in association with bladder outlet obstruction (BOO) secondary to benign prostatic hyperplasia (BPH); however, the two conditions do not invariably coexist. Thus, male LUTS might be due neither to BOO nor prostatic disease. In women, on the other hand, LUTS have usually been equal to OAB and assumed to be caused by detrusor overactivity (DO). Irwin et al., studying 19,000 adult men and women, confirmed that OAB is not solely a female disorder, with the prevalence in both sexes being around 12% and increasing with age[9]. They also found that the prevalence of storage LUTS (suggestive of OAB) was twice as common as voiding LUTS among men. As pointed out by Patel and Chapple, these findings bring into question many of the existing epidemiological data that have been obtained from studies that relied on the assumption that LUTS in men equates with BPH or BOO[10]. Appreciation of a male-specific pattern should lead to a paradigm shift in our understanding of LUTS in men, and its relationship to OAB and DO (see below). LUTS are a nonsex-specific, no-organ-specific group of symptoms that are sometimes age related and progressive, and a broader clinical perspective has been advocated: all LUTS should be treated, not just selected symptoms[11]. There is a need for increased education and awareness regarding LUTS, its causes, and associated comorbidities.

## URGENCY

Urgency, defined by the ICS as "the complaint of a sudden compelling desire to void, which is difficult to defer"[1], is a key symptom of OAB. According to the ICS definition, urgency alone would be sufficient to define OAB. However, according to Irwin et al., only a small proportion of men (9%) and women (8%) with OAB reported urgency only; thus, most of the investigated OAB patients (n = 1434) had more than one symptom[12]. Due to its primary role in defining the OAB syndrome, it is important for clinicians/researchers to have a reasonable understanding of the definition of urgency. However, there is no agreement on whether urgency is an all-or-none abnormal sensation[5], a continuum, or an extreme form of a normal sensation, the "desire to void"[13,14]. Urgency is still often confused with urge and distinguishing urgency from urge has been proposed to clarify the sensory experience perceived by the patient, i.e., urge describing a varying continuum of normal bladder sensation and urgency reflecting a sudden abnormal bladder sensation associated with a compelling desire to void. Fear of leakage, or pain due to a full bladder, were included in previous definitions, but have been omitted from the 2002 ICS

report, despite that these may be important components from the patients' points of view. The definition implies that the sensation is difficult to defer, and thus urgency will persist until the individual either voids voluntarily or an incontinence episode results. Even if the sensation should be "difficult to defer", the possibility to do so is obviously there. This is difficult to reconcile with the view that urgency is a yes or no phenomenon ("the switch is on or off")[5].

It has been suggested that urgency is the primary driver of all symptoms of OAB[5]. However, Yamaguchi et al. found that the occurrence of urgency did not support this concept[15]. In contrast, they found that the urge sensation was markedly increased at any given bladder volume in patients with OAB compared with the normal subjects. This "hypersensitivity" was observed in the patients regardless of urgency episodes. They therefore hypothesized that OAB may be more accurately defined as a hypersensitivity disorder rather than a syndrome characterized primarily by urgency. Homma suggested a new syndrome, "hypersensitive bladder syndrome (HSB)", which should function as a counter-concept of the OAB syndrome[2]. Both syndromes should be parts of a more inclusive syndrome, "frequency/urgency syndrome". Urgency in this context should mean the strong urge to void or the pressing need to void. Urgency in OAB is characterized by sudden onset and/or fear of leakage, while urgency in HSB is of a persistent nature and associated with the fear of pain. Overactive bladder syndrome with leakage (OAB wet) is the typical overactive bladder syndrome. Painful bladder syndrome is the typical hypersensitive bladder syndrome and interstitial cystitis is a representative disease-causing HSB.

The mechanisms behind urgency have not been clarified. There may be defects in afferent signaling resulting from disturbances in urothelial signaling mechanisms (see below), changes in afferent nerves, and in central processing of afferent information. Sensation and micturition in both humans and animals occur in response to afferent signals from the lower urinary tract, and are controlled by neural circuits in the brain and spinal cord. Normally, these circuits coordinate the activity of the smooth muscle in the detrusor and urethra with that of striated muscle in the urethral sphincter and pelvic floor. The reflexes involved are believed to act as on-off switches to shift the lower urinary tract between two modes of operation: storage and voiding[16].

Three distinct sensations have been described at gradual bladder filling during cystometry, i.e., a first sensation of filling, a first desire to void, and a strong desire to void[17]. Wyndaele reported that the first sensation of bladder filling, which is probably only perceived during artificial bladder filling, is weak and not constant[17]. It was suggested that this sensation is dependent on cortical fluctuation in the appreciation and interpretation of afferenty input. It has been shown that impulses related to the desire to void course through the pelvic nerves and those for sensation of a full bladder course through the pudendal nerves. The sense of imminent micturition most probably resides in the urethra and the desire to void comes from stretching the bladder wall. Even if the relation between a strong desire to void, recorded during cystometry[17,18], and the symptom of urgency experienced by OAB patients has not been established, it is generally assumed that changes in these afferent mechanisms may be associated with LUTS, including OAB and DO.

The central handling of afferent information has been studied using different methods, including positron emission tomography (PET). In a PET study, Athwal et al. observed an increased activity in the periaqueductal gray (PAG) matter with increasing bladder volume, and also in the midline pons, in the mid-cingulate cortex, and bilaterally in the frontal lobe area[19]. They interpreted their findings as supporting the hypothesis that PAG receives information about bladder fullness and relays this information to areas involved in the control of bladder storage. They also suggested that the network of brain regions involved in modulating the perception of the urge to void is distinct from that associated with the appreciation of bladder fullness. To assess urge, these authors used a 0–4 perceptual scale where 0 = no sensation, 1 = first sensation, 2 = first urge to void, 3 = strong urge, and 4 = uncomfortable urge. It may be questioned whether uncomfortable urge equals urgency as defined by the ICS or experienced by patients suffering from OAB.

As reflected by the many ways of assessing urgency[6], it is a symptom that is difficult to define, communicate, and measure in a reliable and valid way. Since urgency is the cornerstone of OAB, it is important to study this symptom further in order to achieve a definition that is scientifically acceptable, and also make it possible to measure and communicate to patients.

## FREQUENCY AND NOCTURIA

According to the ICS report, increased daytime frequency is defined as the complaint by the patient who considers that he/she voids too often during the day[1]. This is different from the definitions used in many population-based studies of OAB where frequency has been defined as either eight or more micturitions/day (day = daytime) or eight or more micturitions per 24 h (day = 24 h). Nocturia, according to the ICS, is the complaint that the individual has when he/she wakes at night one or more times to void. In this context, day means from the time of waking in the morning to the time of going to bed at night, and night means from the time of going to bed at night to waking in the morning. Increased daytime frequency is thus a subjective complaint and, therefore, it is not surprising that the number of voids in OAB patients shows a wide variation. If the ICS definition had been used in clinical trials, it is doubtful that it would have been useful as a primary outcome parameter. This is supported by the finding that 75% of men and 60% of women with ICS defined frequency as voiding eight or fewer times a day, most of them five to eight times a day[12].

Nocturia is defined as the number of voids recorded during a night's sleep; each void is preceded and followed by sleep[1]. It is highly prevalent and among the most bothersome LUTS[20]. Nocturia is thus considered to exist if the patient awakes even once during sleep and goes to the toilet. Homma criticized the lack of symmetry in both the term and definition between "daytime frequency" and nocturia[2]. Frequent urination during the daytime is defined by the explanatory term "increased daytime frequency", but frequent urination during the night-time is defined by the single word "nocturia" and not by "increased night-time frequency". Furthermore, with regard to the definition, increased daytime frequency is based on the patient's complaint, while nocturia is based on the number of voids (one or more times). Homma suggested that increased frequency during the day should be called increased daytime frequency and if it occurs during the night, it should be termed increased night-time frequency, the latter term thus replacing nocturia[2].

## OVERACTIVE BLADDER (OAB)

OAB is defined by the ICS as urgency, with or without urgency incontinence, usually with frequency and nocturia[1], in the absence of infection or other obvious pathology. The OAB syndrome can also be described as the urgency syndrome or urgency/frequency syndrome[1,21]. Even if the term has been criticized for serving commercial purposes rather than practical clinical interests, and may have disadvantages from a scientific point of view, it has had a great impact and has now been widely accepted by patients, clinicians, and drug industry.

The prevalence of OAB varies not unexpectedly by the definition. OAB defined as any of the symptoms of urgency, frequency, or urgency incontinence revealed a prevalence of 16–17% of the western population[22,23]. This figure may be an overestimation, and in the study of Irwin et al.[9] applying the ICS 2002 definition, the figure was 12%, similar for men and women. Still, this figure does not reflect the number of patients desiring treatment.

According to the ICS definition, urgency is necessary for the diagnosis of OAB and, theoretically, the only required symptom. However, most patients with OAB also have symptoms of frequency, incontinence, and/or nocturia[12]. It is well established that OAB increases with age. Across all age groups, OAB without urgency incontinence was more common in men than in women. Thus, in women, the OAB prevalence with (OAB wet) and without (OAB dry) urge incontinence was similar (9.3 and 7.6%, respectively). In men, the prevalence of OAB dry was higher than that of OAB wet (13.4 and 2.6%, respectively). Both types of OAB had a significant negative impact on the patients' quality of life[23].

It is important to underline that OAB is a diagnosis based on symptoms and that it is not synonymous with DO (see below). Caution should be exerted particularly when OAB is used in preclinical contexts. There are, for obvious reasons, no animal OAB models, only models of DO.

## DETRUSOR OVERACTIVITY (DO)

According to the ICS, DO is "a urodynamic observation characterized by involuntary detrusor contractions during the filling phase which may be spontaneous or provoked"[1]. Depending on cause, DO may be neurogenic when there is a relevant neurological condition or idiopathic when there is no defined cause.

DO may be associated with OAB in many, but not all, cases[24,25,26,27]. Hashim and Abrams found that, overall, 83% of patients with DO had symptoms of OAB and 64% of patients with OAB had DO[26]. Since it is possible to have asymptomatic DO, Abrams suggested that DO should be categorized as symptomatic or asymptomatic[28]. In the study of Aschkenazi et al., 70% of 383 women with proven DO, urgency, frequency, and urinary incontinence were not found to be statistically significantly associated with DO[27]. The only risk factors that could predict DO with fair diagnostic accuracy were age and nocturia equal to or more than two voids per night. In men, there is a strong correlation between LUTS/OAB (storage LUTS) and DO[29]. However, even so, the relationship between these two entities is unclear and the correlation per se is not enough to support the view that DO is the cause of OAB.

## CONCLUSIONS

The 2002 report of the Standardization Sub-committee of the ICS[1] is considered to be a great improvement towards the standardization of LUT terminology. It is natural and desirable that all suggested definitions are subject to criticism, and thus it is important that discussions for improvement of the existing terminology continue. However, frequent changes of definitions may have detrimental effect not only on research, leading to difficulties in comparing results from different studies, but also may have negative consequences for diagnosis and management of patients with LUT disorders. Continued education and awareness regarding LUTS will hopefully lead to improved management of these symptoms.
